# Nuclear factor I-A represses expression of the cell adhesion molecule L1

**DOI:** 10.1186/1471-2199-10-107

**Published:** 2009-12-14

**Authors:** Tanja Schneegans, Uwe Borgmeyer, Moritz Hentschke, Richard M Gronostajski, Melitta Schachner, Thomas Tilling

**Affiliations:** 1 Zentrum für Molekulare Neurobiologie, Universitätsklinikum Hamburg-Eppendorf, D-20246 Hamburg, Germany; 2 Institut für Med. Mikrobiologie, Virologie und Hygiene, Universitätsklinikum Hamburg-Eppendorf, D-20246 Hamburg, Germany; 3 Department of Biochemistry, State University of New York at Buffalo, Buffalo, NY 14214, USA; 4 Developmental Genomics Group, New York State Center of Excellence in Bioinformatics and Life Sciences, Buffalo, NY 14203, USA; 5 Keck Center for Collaborative Neuroscience, Rutgers University, Piscataway, NJ 08854, USA; 6 Department of Cell Biology and Neuroscience, Rutgers University, Piscataway, NJ 08854, USA; 7 Center for Neuroscience, Shantou Medical College, 22 Xin Ling Road, Shantou 515041, PR China

## Abstract

**Background:**

The neural cell adhesion molecule L1 plays a crucial role in development and plasticity of the nervous system. Neural cells thus require precise control of L1 expression.

**Results:**

We identified a full binding site for nuclear factor I (NFI) transcription factors in the regulatory region of the mouse *L1 *gene. Electrophoretic mobility shift assay (EMSA) showed binding of nuclear factor I-A (NFI-A) to this site. Moreover, for a brain-specific isoform of NFI-A (NFI-A bs), we confirmed the interaction *in vivo *using chromatin immunoprecipitation (ChIP). Reporter gene assays showed that in neuroblastoma cells, overexpression of NFI-A bs repressed L1 expression threefold.

**Conclusion:**

Our findings suggest that NFI-A, in particular its brain-specific isoform, represses *L1 *gene expression, and might act as a second silencer of L1 in addition to the neural restrictive silencer factor (NRSF).

## Background

Neural adhesion molecules of the immunoglobulin superfamily mediate cell-cell recognition by homo- or heterophilic Ca^2+^-independent cell surface interactions [1]. L1, a member of this family, promotes neurite outgrowth and fasciculation, and is involved in axonal pathfinding, neuronal migration, regeneration and synaptic plasticity [1,2]. Targeted ablation of L1 in mice leads to hydrocephalus, corpus callosum hypoplasia, and malformation of the corticospinal tract resembling mutations in the human *L1 *gene that result in an X-linked recessive neurological disorder called X-linked hydrocephalus, MASA syndrome or spastic paraplegia type I (SPG1) (reviewed by [1]). These observations in mice and man point to a key role of L1 in development of the nervous system.

In the control region of the mouse *L1 *gene, a neural restrictive silencer element (NRSE) was identified which is responsible for the neuronal expression of L1 during embryonic development and serves as a tissue-specific silencer and enhancer in postnatal animals [3,4]. Moreover, the transcription factors Pax-6, Hoxa-1, and Barx2 bind to the murine *L1 *gene regulatory region, and Pax-6 activates mouse *L1 *gene expression [5,6]. More recent studies have identified two additional activators of L1 transcription, LEF-1/TCF in human colorectal cancer cells [7], and KLF7 in olfactory sensory neurons [8]. Nuclear factor I-A (NFI-A), a member of the nuclear factor I (NFI) family of site-specific transcription factors, is a good candidate for controlling L1 transcription, as it regulates the expression of several neural proteins and thereby governs development of the central nervous system in mice and men (reviewed by [9,10]). In the present study, we tested whether NFI-A binds to the murine *L1 *gene regulatory region and influences *L1 *gene expression.

## Methods

### Plasmid constructs

The L1 reporter plasmid L1-11 [3], a kind gift from Dr. P. Kallunki (H. Lundbeck A/S, Valby, Denmark), contains 2943 bp upstream of exon 1 of the mouse *L1 *gene, exon 1, intron 1, exon 2 with the luciferase cDNA inserted to replace the L1 start codon by the luciferase start codon, intron 2 including the neural restrictive silencer element, exon 3, intron 3 and exon 4. The NFI-A expression plasmid pCHNFI-A has been described previously [11] and expresses the hemagglutinin (HA) epitope-tagged [12] murine ortholog of chicken NFI-A1.1 [13], which, in this paper, is called "standard isoform" due to its widespread expression in various tissues of adult mice [11]. To express the brain-specific isoform of mouse NFI-A [14] in an HA epitope-tagged form, pCHBNFI-A was used, which was constructed analogously to pCHNFI-A. A plasmid which expresses brain-specific NFI-A lacking most of the activation domain (pCHBNFI-Am) was created by digesting the parental vector with *Bst*XI and *Kpn*I. 3' overhangs were removed by incubation with Platinum Pfx DNA Polymerase (Invitrogen, Karlsruhe, Germany) at 68°C for 15 min, and the resulting blunt ends were ligated using the Rapid DNA Ligation Kit (Roche, Mannheim, Germany). For ChIP experiments, Myc-tagged expression constructs for the standard and brain-specific NFI-A isoforms were made by cutting out the HA tag with *Not*I and *Sfi*I from pCHNFI-A and pCHBNFI-A, respectively. Two oligonucleotides, NFI-Myc 1 (GGCCGCTATGGAACAAAAACTCATCTCAGAAGAGGATCTGCAC) and NFI-Myc 2 (CAGATCCTCTTCTGAGATGAGTTTTTGTTCCATAGC) (Metabion, Martinsried, Germany), which contain the coding sequence of the Myc epitope combined with Kozak box, start codon and the compatible nucleotide overhangs, were annealed. The resulting double-stranded oligonucleotide was added to a 5-10 fold excess of the respective *Not*I/*Sfi*I-digested NFI-A expression vector, and ligated using the Rapid DNA Ligation Kit.

The CMX expression plasmid coding for β-galactosidase [15] was kindly provided by Dr. R. M. Evans (Salk Institute, La Jolla, CA, USA).

### Cell culture

Mouse neuroblastoma cells (N2A) were maintained in Dulbecco's modified Eagle's medium (DMEM) containing 10% fetal calf serum, 1 mM sodium pyruvate, 2 mM L-glutamine, and antibiotics (100 units/ml penicillin and 100 μg/ml streptomycin). For reporter gene assays, N2A cells were grown in Opti-MEM without phenol red (Invitrogen, Karlsruhe. Germany) supplemented with 5% fetal calf serum, 200 units/ml of penicillin G, and 200 μg/ml streptomycin. Chinese hamster ovary cells (CHO) were grown in Glasgow minimum essential medium (GMEM), which contained 10% fetal calf serum, 4 mM L-glutamine, and antibiotics (100 units/ml penicillin and 100 μg/ml streptomycin). All cell types were grown at 37°C under a 5% CO_2 _atmosphere.

### Electrophoretic mobility shift assays (EMSAs)

For EMSAs, HA-NFI-A constructs were expressed in CHO cells. Cells were plated onto 90-mm dishes in CHO culture medium. After reaching confluence, cells were washed once with CHO culture medium without serum and antibiotics. Transfections were performed with Lipofectamine (Invitrogen) according to the manufacturer's instructions. Per dish, 13 μg of the respective plasmid were transfected with 26 μl Lipofectamine and 39 μl Plus Reagent (Invitrogen). Three h later, the transfection reaction was stopped by addition of 6.5 ml CHO culture medium. 24 h after transfection, cell monolayers were washed once with PBS and harvested by scraping into PBS supplemented with Complete protease inhibitor (Roche). Cells were pelleted by spinning for 10 min at 4°C (300 × g), the supernatant was removed and the pellet resuspended in low salt lysis buffer (20 mM HEPES pH 7.8, 100 mM NaCl, 5 mM DTT, 1 × Complete protease inhibitor (Roche)). The cell suspension underwent three freeze-thaw cycles in dry ice/ethanol. Cell debris were removed by centrifugation (15 min, 4°C, 15000 × g), and the supernatant was used for DNA binding analysis. The electrophoretic mobility shift experiments were essentially performed as described [16]. In brief, single-stranded oligonucleotides were purchased (Metabion) and annealed, yielding double-stranded oligonucleotides with 5' overhangs. Double-stranded oligonucleotides were labeled with α-^32^P-dCTP using Klenow polymerase (Roche). Binding reactions were performed in a total volume of 10 μl consisting of 20 mM HEPES pH 7.8, 100 mM NaCl, 2 mM MgCl_2_, 7 mM DTT, 0.5 μg Cot-1 DNA, and 3 μl of cell lysate. Complete protease inhibitor was added according to the manufacturer's specifications (Roche). Binding reactions were incubated for 20 min followed by the addition of 2 μl of the labeled oligonucleotides, and incubated further for 20 min at room temperature. For the supershift, 1 μl of undiluted anti-HA (clone 12CA5, Roche) was added before loading, and incubated for another 20 min. In the case of competition experiments, radioactively labeled oligonucleotides were mixed with a 5-100 fold molar excess of the respective unlabeled oligonucleotides before being added to the binding reaction. Complexes were resolved by nondenaturing PAGE, and dried gels were exposed to BioMax MR film (Kodak, Stuttgart, Germany).

Oligonucleotides used were as follows: L, 5'-gctatTTGGCTTGGTGCCAAgcatc-3'; Lc, 5'-gctatTT**C**GCTTGGTGCCAAgcatc-3'; N, 5'-aggtCTGGCTTTGGGCCAAgagccgc-3'; Nc, aggtCT**C**GCTTTGGGCCAAgagccgc-3'; SIS, 5'-agcttaccagaaggtcaaggtcaaatgaagctagct-3' Sequences corresponding to the NFI binding site are capitalized; mutations in the negative control oligonucleotides are shown in boldface. The sequence of one strand is shown after the fill-in reaction.

### Western Blot

In order to check HA-NFI-A expression by Western Blot, transfection of CHO cells was performed as described for EMSAs. However, 6-well plates were used, with DNA amounts and volumes of media and reagents adjusted accordingly. 24 h after transfection, cells were lysed by incubating in Ripa buffer (50 mM Tris pH 7.4, 150 mM NaCl, 1 mM EDTA, 0.5% (w/v) NP-40, 1 × Complete protease inhibitor (Roche)) for 1 h at 4°C. Cell debris were removed by centrifugation (15000 × g, 4°C, 10 min). The supernatant was mixed with sample buffer and boiled for 5 min. Proteins were separated by SDS-PAGE, transferred to a nitrocellulose membrane, and immunologically detected as described (Kalus *et al. *2003). Anti-HA (from mouse, clone 12CA5, Roche) was used as primary antibody (1:400 in 4% milk powder in Tris-HCl-buffered saline (pH 7.3)). Horseradish peroxidase-conjugated goat anti-mouse secondary antibody (Dianova, Hamburg, Germany) was applied at a 1:10,000 dilution in 4% milk powder in Tris-HCl-buffered saline (pH 7.3). Detection of Myc-NFI-A st and Myc-NFI-A bs expression in N2A cells was performed following the same protocol, using anti-Myc (from mouse, clone 9E10, Santa Cruz, Heidelberg, Germany) as a primary antibody. Transfection of the respective plasmids was performed as described under "Chromatin immunoprecipitation assay". However, 6-well plates were used, with DNA amounts and volumes of media and reagents adjusted accordingly. To check for the amount of protein loaded per lane, an anti-GAPDH antibody was applied (anti-GAPDH from rabbit, Cell Signalling, mAb 14C10, 1:1000; followed by anti-rabbit-IgG-HRP, 1:20,000).

### Chromatin immunoprecipitation assay (ChIP)

Myc-tagged NFI-A expression for ChIPs was carried out in N2A cells, cultivated on 90-mm dishes until confluency. 13 μg of the respective plasmid were transfected with 26 μl Lipofectamine and 39 μl Plus Reagent (Invitrogen) per dish according to the manufacturer's instructions. 48 h later, ChIP analysis was essentially performed as described [17]. For reduction of non-specific background, 0.5 μg anti-HA was applied per precipitation. Specific precipitation was achieved using anti-Myc. DNA fragments were purified with the MinElute PCR purification kit (Qiagen, Hilden, Germany). Subsequent PCR was carried out with the primers ChIP fw, GGAGTTCAAATGCCTTAACATGA, and ChIP rev, CTGGATGCCCTCAATAAATTCAT. For negative control amplifications of remote gene loci, the following primers were used: Chst11 fw, TGGAGACAGCCCTCCATAGATGT; Chst11 rev, GATGGCAGTGTTGGATAGCTCCA; Chst8 fw, GTAAACGACTTCTCCTACCGCA; Chst8 rev, GTATTGTCACAGGGACGATGTCCA. Amplification comprised 25 cycles, with the annealing temperature set to 55°C. As a positive control, genomic DNA from C57BL/6J mouse tail cuts was used, which was prepared according to the following protocol: tail cuts were incubated over night at 55°C in 100 μl lysis buffer (0.1 mg/ml proteinase K (Sigma, Taufkirchen, Germany); 50 mM Tris pH 8.0; 50 mM KCl; 2.5 mM EDTA; 0.45% NP-40 and 0.45% Tween-20). The next day, tissue debris were spun down, and the whole supernatant was extracted with an equal volume of PCI (phenol-chloroform-isoamyl alcohol, Biochrom, Berlin, Germany). DNA was precipitated with 2.5 volumes of ethanol and 0.1 volumes of 8 M LiCl. The pellet was washed with 70% ethanol and, after drying, resuspended in 50-100 μl 1 mM Tris pH 8.0.

### Luciferase-based reporter gene assays

For transient transfections, 1 × 10^4 ^mouse neuroblastoma (N2A) cells were seeded in 96-well tissue culture plates (Greiner, Frickenhausen, Germany) and transfected the next day with 2.5 μl Lipofectamine 2000 (Invitrogen) per microgram of DNA. 80 ng reporter plasmid (L1-11), 60 ng of the effector plasmids (pCHBNFI-A or pCHBNFI-Am) or control CMX plasmid coding for β-galactosidase were applied per well. EGFP fluorescence generated by cotransfection of 60 ng pEGFP-C3 (Clontech, Heidelberg, Germany) was determined after 48 hours with a Wallac 1420 Multilabel Counter (PerkinElmer, Wiesbaden, Germany). Luciferase activity was measured with the Bright-Glo Luciferase Assay System according to the manufacturer's instructions (Promega, Mannheim, Germany). Light emission was normalized to the level of EGFP fluorescence activity from the EGFP control plasmid. Transfections were generally performed in septuplicate. Average relative luciferase light units and standard deviations were calculated using the Prism-4 program (GraphPad Software, La Jolla, CA, USA).

## Results

### Identification of a full NFI binding site in the first intron of the mouse *L1 *gene

**Figure 1:**
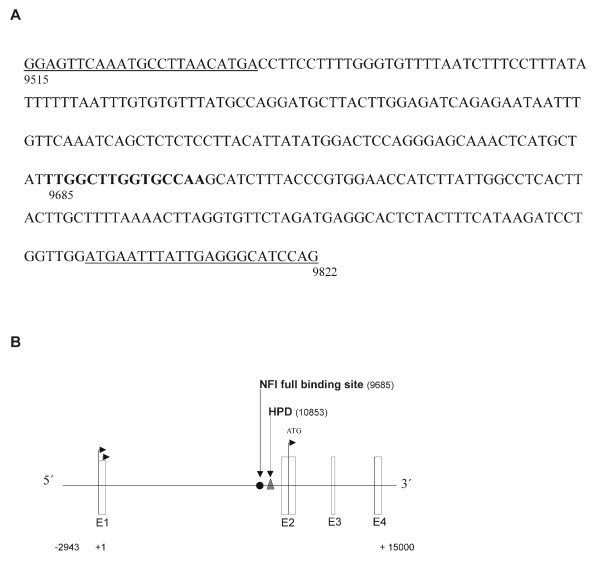
**The regulatory region of the mouse *L1 *gene contains a full NFI binding site**. **A**, 15-nucleotide sequence (*boldface*) fully matching the NFI consensus binding motif [9]. Numbering with respect to the first nucleotide of exon 1 according to the mouse *L1 *gene nucleotide sequence deposited in GenBank (accession number NC_000086.6); sequences corresponding to the primers for ChIP analysis are underlined. **B**, The first four exons of the mouse *L1 *gene are indicated by boxes labeled E1-E4. *Arrows*: transcription initiation sites; *ATG*: translation start; *filled circle*: full NFI binding site; *triangle*: homeodomain and Pax protein binding site (HPD).

To ask whether NFI-A binds to the murine *L1 *gene regulatory region, we analyzed the nucleotide sequence of this region [3,5,6] for NFI binding sites, using the Lasergene (DNASTAR Inc., Madison, WI) software suite. We identified the sequence TTGGCTTGGTGCCAA, which fully matches the NFI consensus motif, TTGGC(N)_5_GCCAA [9], in intron 1, located 1341 bp upstream of the translation start codon in the second exon (Fig. 1). The same part of the mouse *L1 *gene regulatory region also contains a previously identified DNA element recognized by Pax-6 and by additional homeodomain proteins [6] (see Fig. 1B). In addition to the full binding site, we found eight half binding sites for NFI proteins located within the 2400 bp immediately upstream of the full site (not shown).

### NFI-A binds to the regulatory region of the mouse *L1 *gene *in vitro*

**Figure 2:**
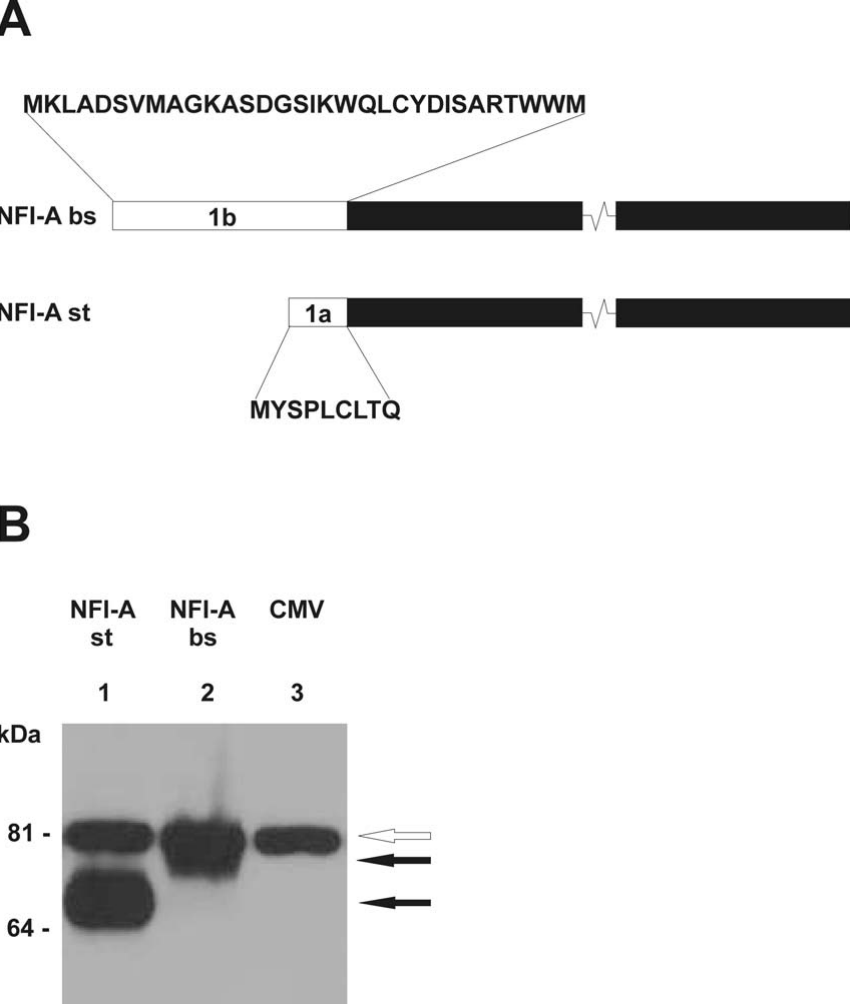
**Expression of HA-tagged NFI-A isoforms in CHO cells**. **A**, Schematic drawing of the brain-specific isoform ("NFI-A bs") and the ubiquitous "standard" isoform ("NFI-A st") of mouse NFI-A. The isoform-specific N-termini (*open rectangles*) are encoded by exons 1a and 1b, respectively, and result from alternative splicing. Amino acid sequences encoded by these exons are given above and below. The major part of the protein (*filled rectangles; not drawn to scale*) is identical in both isoforms. Total length: 532 amino acids (NFI-A bs); 509 amino acids (NFI-A st). **B**, CHO cells were transfected with 2 μg of NFI-A cDNA expression vectors. Lane 1, pCHNFI-A ("standard" isoform); lane 2, pCHBNFI-A (brain-specific isoform); lane 3, mock transfected. Whole cell extracts were analyzed on an 8% SDS-PAGE gel, transferred to nitrocellulose membrane, and probed with anti-HA antibody. *Filled arrows *indicate bands which correspond to the respective HA-tagged NFI-A polypeptides, the *open arrow *shows a nonspecific band.

**Figure 3:**
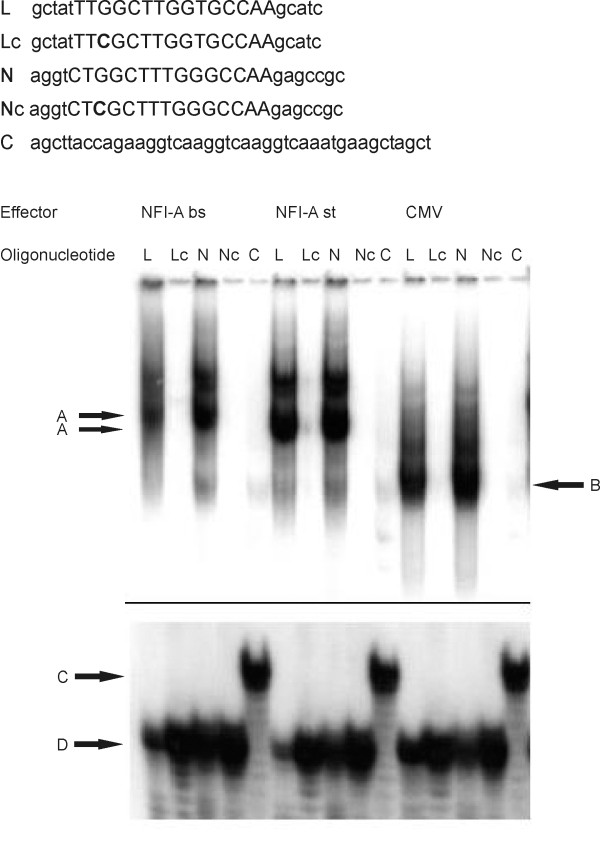
**NFI-A binds to its full binding site in the mouse *L1 *gene *in vitro***. ^32^P-labeled oligonucleotides used for EMSA: L: full NFI binding site in mouse *L1 *gene with flanking sequences; Lc: point-mutated variant of L; N: idealized NFI binding site with flanking sequences [18]; Nc: point-mutated variant of N; C: oligonucleotide without NFI recognition motif (SIS oligonucleotide). NFI binding site sequences are *capitalized*, point mutations are shown in *boldface*. The sequence of one strand is shown after the fill-in reaction. Oligonucleotides were incubated with extracts from CHO cells expressing NFI-A bs or NFI-A st, or from mock-transfected cells (CMV). Only the upper and lower parts of the autoradiogram are shown, the middle section, which did not exhibit any signals, was cut out for reasons of space. *Arrows *A: HA-NFI-A-DNA complexes; *arrow *B: complexes of endogenous NFI and DNA; *arrows *C and D: free oligonucleotide DNA. Note that that the free SIS oligonucleotide migrates slightly slower (*arrow *C) than the other free oligonucleotides (*arrow *D) due to its higher molecular weight.

**Figure 4:**
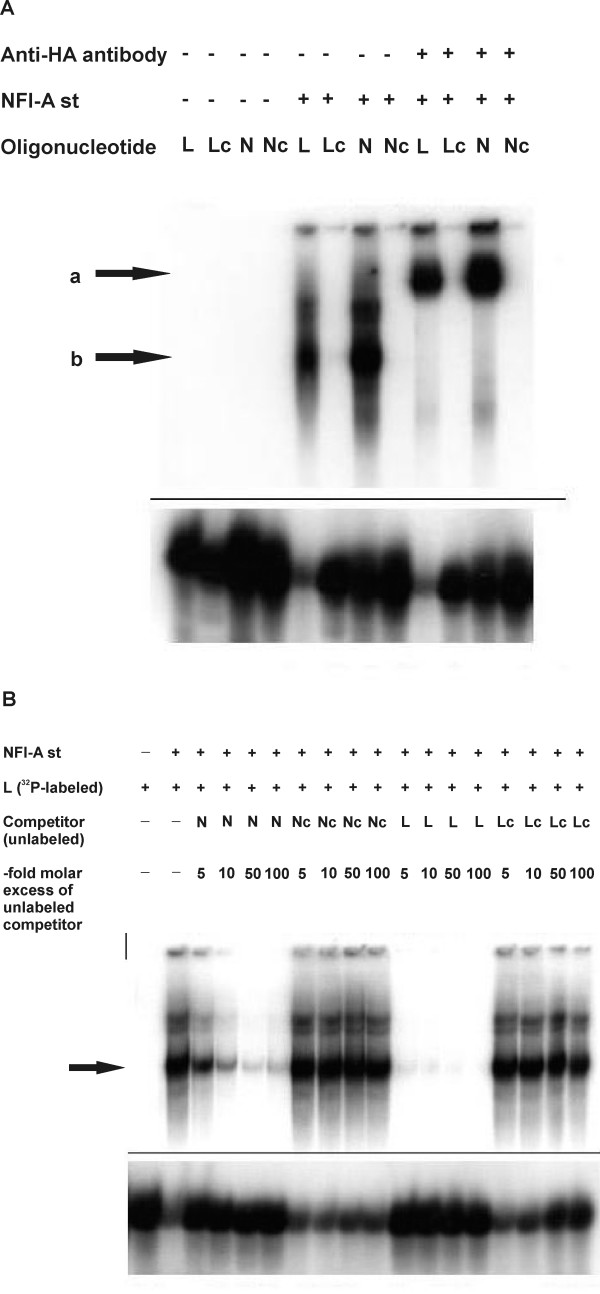
***In vitro *binding of NFI-A to its full binding site in the mouse *L1 *gene is confirmed by supershift and competition assays**. EMSAs were performed with ^32^P-labeled oligonucleotides indicated above the autoradiograms (for a description, see Fig. 3). In both images (**A-B**), only the upper and lower parts of the respective autoradiogram are shown, the middle sections, which did not exhibit any signals, were cut out for reasons of space. **A**, Supershift experiment. NFI-A st "+": oligonucleotides were incubated with extracts from CHO cells transfected with pCHNFI-A (NFI-A st); NFI-A st "-": oligonucleotides were not pre-incubated with cell extracts (negative control). Where indicated, anti-HA antibody was added. *Arrow a *points at anti-HA-antibody/NFI-A/DNA super-shifted complexes, while *arrow ***b **indicates NFI-A/DNA complexes. **B**, Competition analysis with the radioactively labeled NFI binding site from the mouse *L1 *gene regulatory region (L) and various unlabeled competitor oligonucleotides (N, Nc, L, Lc; see Fig. 3 for a description). The ^32^P-labeled L oligonucleotide was incubated with extracts from CHO cells transfected with pCHNFI-A (NFI-A st) in the presence of different molar excesses of the unlabeled competitor oligonucleotides as indicated above the autoradiogram. A "-" indicates that oligonucleotides were not pre-incubated with cell extracts (negative control). The *arrow *points at the main band representing NFI-A-DNA complexes.

To assess binding of NFI-A to the full NFI recognition motif in the *L1 *gene regulatory region *in vitro*, electrophoretic mobility shift assays (EMSAs) were performed, using both the ubiquitous "standard" isoform of NFI-A (NFI-A st) and a brain-specific isoform (NFI-A bs) [14]. Their unique N-termini of 32 (NFI-A bs) and 9 amino acids (NFI-A st) are generated by alternative splicing [9] (Fig. 2A). Both HA-tagged isoforms were expressed in CHO cells (Fig. 2B). Cell extracts were incubated with oligonucleotides containing either the newly identified full binding site in the mouse *L1 *gene (L), or an idealized NFI binding site (N, [18]) (Fig. 3). To control for binding specificity, a G→C point mutation eliminating an essential GN7 contact for NFI was introduced into both oligonucleotides, yielding Lc and Nc, respectively (Fig. 3). Both NFI-A st and NFI-A bs expression caused a significant shift of the L and N oligonucleotides (Fig. 3). Neither the Lc nor the Nc oligonucleotide were shifted, indicating that the guanosine in position 3 of the motif is critical for NFI-A binding and that the complex is formed by sequence-specific interaction of NF1-A with both elements. Moreover, no gel shift was observed for the unrelated SIS oligonucleotide (C) [16], which does not contain an NFI recognition motif. Extracts without forced NFI-A expression ("CMV", Fig. 3, label B) gave rise to faster mobility complexes. These complexes most likely represent endogenous NFI isoforms as indicated by the lack of binding to the mutant Lc, Nc and C oligos. The heterogeneity of these endogenous complexes and their reduction in the transfected samples is likely due to the known alternative splicing of NFI proteins and the ability of NFI proteins to form both homo- and hetero-dimers *in vivo*, respectively. Specific binding was confirmed with an anti-HA supershift experiment (Fig. 4A).

In competition experiments, the L oligonucleotide was more effective than the N oligonucleotide (Fig. 4B), implicating a higher affinity of NFI-A to the site in the *L1 *gene as compared to the idealized consensus site. The point-mutated oligonucleotides Lc and Nc were unable to compete with the intact binding motifs. We conclude that, *in vitro*, NFI-A binds to its recognition site in the first intron of the mouse *L1 *gene, and that this interaction is tight and specific.

### The brain-specific isoform of NFI-A binds to the regulatory region of the mouse *L1 *gene *in vivo*

**Figure 5:**
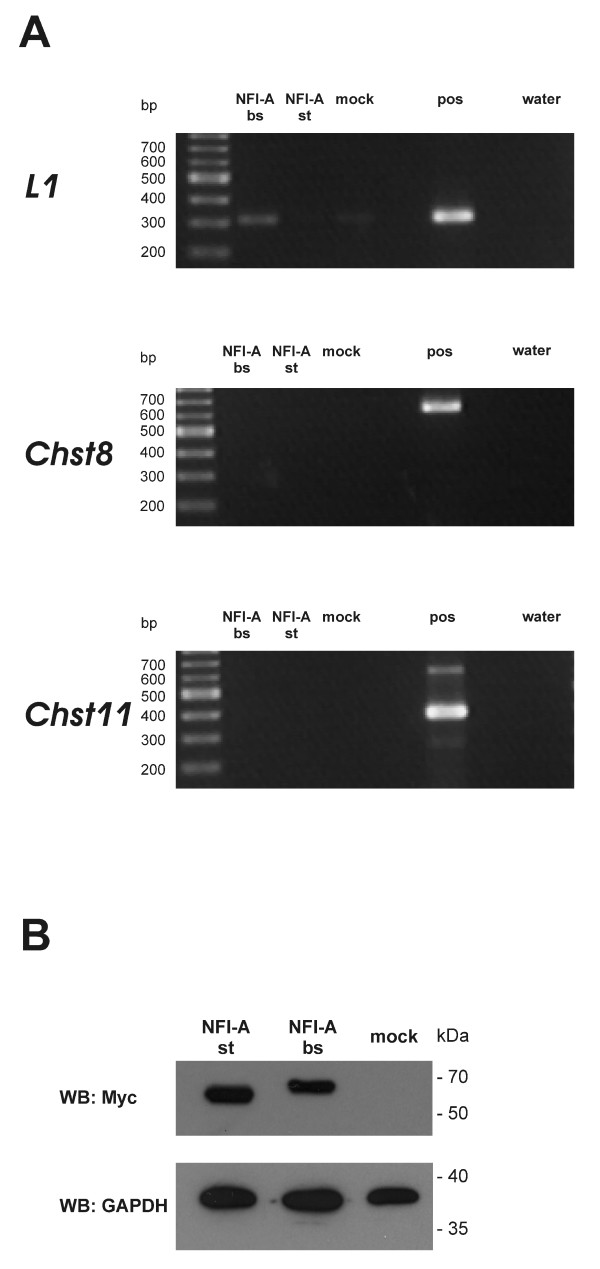
**NFI-A binds to the *L1 *gene *in vivo***. **A**, ChIP analysis of N2A cells expressing Myc-NFI-A. ***L1***, NFI-A-DNA complexes in the region of the *L1 *gene containing the NFI full binding site were assayed by PCR using primers indicated in Fig. 1A. *Lanes: NFI-A bs*, precipitation from Myc-NFI-A bs-transfected cells; *NFI-A st*, precipitation from Myc-NFI-A st-transfected cells; *mock*, precipitation from mock-transfected cells; *pos*, positive control (mouse genomic DNA as PCR template); *water*, water control (without PCR template). ***Chst8 ***and ***Chst11***, To further confirm the specificity of ChIP analysis, ChIP samples were analyzed by PCR using primers amplifying parts of the mouse *Chst8 *and *Chst11 *genes, respectively. Note the absence of PCR signals for *Chst8 *and *Chst11 *in the ChIP sample lanes. **B**, Expression of Myc-tagged NFI-A isoforms in N2A cells. N2A cells were transfected with 4.8 μg of NFI-A cDNA expression vectors to express Myc-NFI-A st or Myc-NFI-A bs, respectively, or with 4.8 μg pCMX plasmid ("mock"). 48 h after transfection, cell were lysed and whole cell extracts were analyzed on a 10% SDS-PAGE gel, transferred to nitrocellulose membrane, and probed with anti-Myc antibody for detection of recombinant Myc-NFI-A and with anti-GAPDH antibody to check for loading of comparable protein amounts.

To ask whether the interaction between NFI-A and its binding motif in the mouse *L1 *gene regulatory region is seen *in vivo*, we performed a chromatin immunoprecipitation (ChIP) analysis. Mouse neuroblastoma (N2A) cells, which express endogenous L1 [19], were transfected with Myc-tagged NFI-A bs or Myc-tagged NFI-A st, respectively. Precipitation was carried out with an anti-Myc antibody, and PCR primers were chosen which specifically amplify the region of the *L1 *gene flanking the NFI full binding site (Fig. 1A). Fig. 5A (upper panel, *L1*) shows the outcome of a representative experiment: whereas binding of the brain-specific NFI-A isoform (NFI-A bs) to the *L1 *regulatory region could be confirmed, no signal was obtained for the ubiquitous isoform (NFI-A st). The same result was observed in two further ChIP assays, and only in one experiment, weak binding of NFI-A st was detected. Only weak PCR signals were observed for mock-transfected cells. As an additional negative control, segments of the mouse *Chst8 *and *Chst11 *genes were amplified by PCR. No signals were observed when ChIP precipitates were used as a template (Fig. 5A, middle panel and lower panel), confirming the specificity of NFI-A bs binding to the mouse *L1 *gene. Western blot analysis revealed the presence of comparable amounts of both NFI-A isoforms in transfected N2A cells (Fig. 5B). Thus, the brain-specific isoform of NFI-A directly interacts with the endogenous *L1 *gene regulatory region in a neuronal cell line.

### NFI-A represses activity of the *L1 *gene in mouse neuroblastoma cells

**Figure 6:**
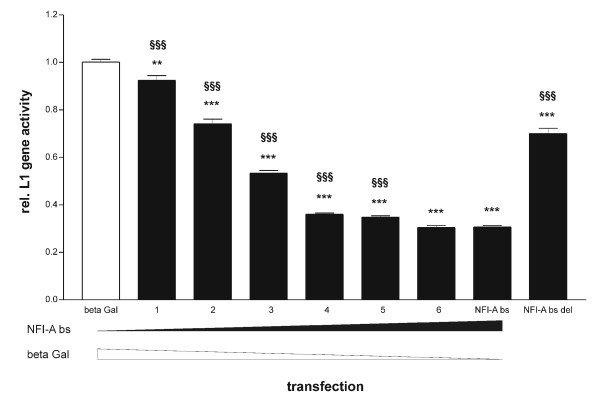
**NFI-A represses mouse *L1 *gene expression**. Results of luciferase-based reporter gene assays in N2A cells transfected with L1-11. The L1 reporter plasmid L1-11 contains 2943 bp upstream of exon 1 of the mouse *L1 *gene, exon 1, intron 1, exon 2 with the luciferase cDNA inserted to replace the L1 start codon by the luciferase start codon, intron 2 including the neural restrictive silencer element, exon 3, intron 3 and exon 4. beta Gal: coexpression of beta-galactosidase (negative control) (*n *= 98); NFI-A bs: coexpression of NFI-A bs (*n *= 91); NFI-A bs del: coexpression of NFI-A bs del (*n *= 35). For titration of beta Gal with NFI-A bs, the beta Gal expression plasmid was gradually replaced with the NFI-A bs expression plasmid at the ratios indicated below, leaving the total amount of transfected DNA constant. "1", bs:Gal = 1:59 (*n *= 28); "2", bs:Gal = 1:11 (*n *= 35); "3", bs:Gal = 1:5 (*n *= 49); "4", bs:Gal = 1:2 (*n *= 21); "5", bs:Gal = 1:1 (*n *= 49); "6", bs:Gal = 2:1 (*n *= 21). Data were acquired from 3 (bs:Gal = 1:2) to 9 (beta Gal, NFI-A bs) independent experiments. For normalization, luciferase activity was divided by the fluorescence of coexpressed EGFP. The relative luciferase activity of the beta Gal-transfected samples was set to 1. **, *p *< 0.01 vs. beta Gal; ***, *p *< 0.001 vs. beta Gal; ^§§§^, *p *< 0.001 vs. NFI-A bs (two-tailed, unpaired *t*-test).

For a functional analysis, the effect of forced NFI-A bs expression on the activity of a previously described L1-luciferase reporter construct, L1-11 [3], was determined in N2A cells. The brain-specific isoform of NFI-A was chosen because it reproducibly bound to the endogenous *L1 *regulatory region (Fig. 5A), whereas *in vivo *binding of the ubiquitous NFI-A isoform appeared to be weaker. NFI-A bs reduced *L1 *gene activity to less than one-third of control values (Fig. 6, second column from the right side). Repression was concentration-dependent, suggesting a specific effect (Fig. 6). Moreover, we investigated a mutated form of NFI-A bs (NFI-A bs del), which lacked the entire activation/repression domain. NFI-A bs del should therefore be able to bind to DNA, but exhibit a severely reduced ability to activate or repress target genes [9]. In line with these expectations, expression of the truncated NFI-A bs led to only a slight reduction of L1 transcription (Fig. 6, rightmost column). Summarizing, our data show a repressive influence of NFI-A bs on *L1 *gene expression, which can be reverted by impairing NFI-A's trans-regulatory abilities.

## Discussion

The cell adhesion molecule L1 plays a crucial role in mammalian nervous system development, but regulation of its expression at the transcriptional level is only partly understood. Here, using EMSA and antibody supershift experiments, we showed that the site-specific transcription factor NFI-A specifically interacts with a full NFI recognition site in the first intron of the murine *L1 *gene. The interaction is very strong, as shown by competition analysis. This high affinity is in accordance with a previous *in vitro *study, in which the NFI consensus binding motif was determined by PCR-mediated random site selection [20]. We also observed an electrophoretic mobility shift using extracts from cells not transfected with NFI-A expression plasmids, probably caused by expression of endogenous NFI proteins. The respective bands were much weaker when NFI-A was overexpressed, either due to a limiting input or due to a down-regulation of endogenous NFI-A by forced NFI-A expression.

ChIP analysis confirmed that the brain-specific isoform of NFI-A (NFI-A bs) binds to the regulatory region of the mouse *L1 *gene *in vivo *as well. By contrast, we did not observe reproducible binding of the ubiquitous NFI-A isoform (NFI-A st) to this genomic region *in vivo*, although the *in vitro *interaction of NFI-A st with the full binding site in *L1 *was at least as strong as the one of NFI-A bs. This difference might be caused by the different binding reaction conditions in the two assays. Alternatively, post-translational modifications could be a reason for the different behavior of NFI-A st and NFI-A bs in the two assays, in particular phosphorylation, which has been implied in regulating NFI activity by several studies [21-23]. Whereas for EMSA, extracts from CHO cells were used, ChIP was performed using N2A cells. It is thus tempting to speculate that phosphorylation of crucial amino acid residues differs between NFI-A bs and NFI-A st in N2A cells, causing a different affinity to the *L1 *gene regulatory region. Differences in protein phosphorylation or other modifications might also explain why the apparent molecular weights of NFI-A bs and NFI-A st differed slightly more than one would expect from their amino acid composition. Finally, it should be noted that eight half binding sites for NFI proteins can be found in the 2400 bp immediately upstream of the full site. NFI-A binds to such pentanucleotide sequences with a reduced affinity [20].

In order to understand how binding of NFI-A to the *L1 *regulatory region influences L1 expression, we performed reporter gene assays in mouse neuroblastoma (N2A) cells. NFI-A bs caused a reduction in *L1 *gene activity to approx. 30% of control level. In this context, it is noteworthy that only the brain-specific isoform significantly interacted with the endogenous *L1 *gene regulatory region. Moreover, we could nearly abolish repression of L1 transcription by NFI-A bs by deleting NFI-A's transregulatory domain. To our knowledge, these results are the first experimental evidence for a specific role of NFI-A bs in the regulation of a neuronal gene.

How could NFI-A activity at the *L1 *gene regulatory region be regulated in a physiological context? Several studies suggest that NFI activity is modulated by NFI phosphorylation [21-23]. In our case, NFI-A could be inactivated by phosphorylation, leading to enhanced L1 expression. However, there is no direct evidence that phosphorylation affects NFI activity.

Interactions with other site-specific transcription factors might also regulate transactivation/transrepression by NFI-A. NFI proteins physically interact with TTF-1 [24], Oct-1 [25], ski [26], and CBP [27]. Binding of NFI-A to such factors could alter its inhibitory influence on L1 transcription initiation. Remarkably, the HPD element, which is responsible for stimulation of L1 expression by Pax-6, is located in the same part of the *L1 *gene as the full NFI binding site identified in our study [6]. In addition, binding of the homeodomain proteins Barx-2 [6] and Hoxa-1 [5] to the *L1 *gene is also mediated by the HPD element. This might implicate a functional interaction between NFI transcription factors and Pax-6, Barx-2, or Hoxa-1 in regulating L1 expression.

## Conclusions

In summary, our data suggest that NFI-A is a repressor of mouse *L1 *gene activity. A role for NFI-A in regulating L1 expression during development is plausible, as expression of both genes starts around embryonic day 9 in the developing nervous system of the mouse [3,11]. In addition, Kallunki *et al. *[3] have proposed a second silencer element in the first intron of the L1 gene that functions together with the NRSE to regulate L1 expression. NFI-A, binding tightly to its recognition motifs in this intron and repressing L1 expression, could be part of the proposed silencer. It may prevent L1 expression at inappropriate stages or cell types, like glia of the central nervous system, consistent with the recently demonstrated importance of NFI-A for glial cell differentiation [28-31]. As both L1 and NFI-A are crucial for brain development in humans [1,32], further investigations on regulation of L1 expression by NFI-A, for instance cell type-specific analyses of NFI-A deficient mice during development, are likely to significantly extend our knowledge of human brain formation.

## Authors' contributions

TS designed and performed experiments shown in Fig. 2, 3, 4, and 5A and participated in writing the manuscript. UB designed and carried out the reporter gene assays, provided support for EMSAs, and participated in writing the manuscript. MH participated in the ChIP assay. RMG participated in the design of the study, performed the analysis shown in Fig. 1, and participated in writing the manuscript. MS conceived of the study and participated in writing the manuscript. TT conceived of the study, supervised experimental design, participated in the ChIP assay, performed the experiment shown in Fig. 5B and wrote the manuscript. All authors read and approved the final manuscript.
